# Transient hypothyroidism in a neonate following maternal exposure to vinblastine during pregnancy: a case report and review

**DOI:** 10.3389/fped.2024.1464520

**Published:** 2024-12-23

**Authors:** Zubair Amin, Win Thu Aung, Yvonne Peng Mei Ng

**Affiliations:** ^1^ Department of Neonatology, Khoo Teck Puat-National University Children Medical Institute, National University Health System, Singapore, Singapore; ^2^ Department of Paediatrics, Yong Loo Lin School of Medicine, National University of Singapore, Singapore, Singapore; ^3^ Yong Loo Lin School of Medicine, National University of Singapore, Singapore, Singapore

**Keywords:** vinca alkaloids, pregnancy, toxicity, vincristine, fetus, neonate

## Abstract

**Background:**

Vinblastine is a widely used chemotherapeutic agent for various cancers. We report a case of transient congenital hypothyroidism following maternal exposure to vinblastine during the third trimester of pregnancy and propose possible mechanisms of action.

**Method:**

We utilized the CARE guidelines to report the case.

**Case presentation:**

The mother is a 30-year-old previously healthy Malay woman who was diagnosed with Hodgkin lymphoma during her 28th week of pregnancy. She received two cycles of vinblastine monotherapy during her third trimester. She delivered a healthy baby girl at 37 weeks of gestation. Cord blood screening for congenital hypothyroidism and subsequent thyroid function tests showed evidence of congenital hypothyroidism. An ultrasound scan of the baby confirmed the presence of the thyroid gland, but there was no uptake of radionuclide tracer by the thyroid gland, thereby ruling out thyroid dysgenesis or dyshormonogenesis as the plausible cause of hypothyroidism. The baby was treated with replacement thyroxine for 18 months with eventual normal growth and development.

**Conclusion:**

This is the first reported case of transient congenital hypothyroidism following maternal exposure to vinblastine during the third trimester of pregnancy. Although direct causation cannot be established, heightened awareness of neonatal hypothyroidism is recommended after exposure to vinblastine or similar drugs in pregnancy.

## Introduction

Vinblastine, a chemotherapeutic agent closely associated with vincristine, is one of the oldest chemotherapeutic agents. Vincristine and vinblastine are derived from the Madagascar periwinkle plant and are known as vinca alkaloids (1). Vinblastine is a commonly used chemotherapy agent for Hodgkin lymphoma, anaplastic large cell lymphoma, Kaposi sarcoma, ovarian germ cell tumors, and low-grade gliomas (2). Vinblastine is categorized as a Pregnancy Category D drug, indicating that “There is positive evidence of human fetal risk based on adverse reaction data from investigational or marketing experience or studies in humans, but potential benefits may warrant use of the drug in pregnant women despite potential risks” (3). It is on the list of the World Health Organization's (WHO) Essential List of Drugs under the category of cytotoxic medicines (2). Although commonly used in combination with other chemotherapeutic agents, vinblastine can be used as a single agent for the aforementioned indications (4).

We report a case of transient congenital hypothyroidism following maternal exposure to vinblastine during the third trimester of pregnancy and elucidate possible mechanisms of action. We also review the use of selected chemotherapeutic agents used during pregnancy.

## Method

We have utilized the CARE guidelines in reporting this case (5) (Supplementary Material 1). We obtained parental written informed consent and have included the family's perspectives in this manuscript.

## Case presentation

The mother is a 30-year-old previously healthy woman of Malay (Southeast Asian) ancestry. Her thyroid function tests were normal on multiple occasions: twice during her previous pregnancy (3 years before the current one), once during this pregnancy, and twice after the delivery of this child (at 1 and 2 years after the delivery). She underwent no thyroid antibody test as her thyroid function tests were normal. There is no family history of thyroid disorders. She and her husband are non-consanguineous. She was in her third pregnancy, with a 2-year-old healthy child and one previous miscarriage. She developed cervical lymphadenopathy during her 28th week of pregnancy, and radiological imaging revealed an anterior mediastinal mass. She was diagnosed with stage IV Hodgkin lymphoma from a cervical lymph node biopsy. Following the diagnosis, she received two cycles of intravenous vinblastine as a single agent at around 31 and 33 weeks of gestation. She did not receive any radiotherapy nor undergo computed tomography scans during her current pregnancy.

She delivered a baby girl at 37 weeks of gestation via uneventful vaginal birth. The baby's birth weight was 2,865 g (49th percentile), length was 47.5 cm (46th percentile), and occipitofrontal circumference was 32.5 cm (35th percentile) based on the World Health Organization's growth curve. The baby did not present with any malformation. Physical examination, hearing assessment, and screening tests for inborn error of metabolism were normal for the baby. The baby developed neonatal jaundice, which was attributed to mild hemolysis resulting from maternal–baby blood group incompatibility, and was treated with phototherapy. Her full blood count demonstrated no evidence of myelosuppression and showed a high reticulocyte response (reticulocyte count of 11.2%) in response to mild hemolysis. The mother did not breastfeed the baby.

The baby's umbilical cord thyroid-stimulating hormone (TSH), measured as part of the universal newborn screening program in Singapore, was at the higher end of normal at 23.96 mIU/L (reference range: 2.20–25.00 mIU/L), with a normal free thyroxine (FT4) level of 13.5 pmol/L (reference range: 7.5–22.0 pmol/L). A repeat thyroid function test on day 5 of life showed a TSH level of 18.85 mIU/L (reference range: 0.80–5.90 mIU/L) and an FT4 level of 19.4 pmol/L (reference range: 17.4–57.7 pmol/L). On day 21 of life, the repeat TSH level was 24.98 mIU/L (reference range: 0.80–5.90 mIU/L), with the FT4 level of 13.0 (reference range: 9.5–17.8 mIU/L). As the TSH value remained persistently high and was increasing, the baby was started on oral thyroxine replacement at 12.5 μg per day (4.6 μg/kg/day based on weight of 2.7 kg) for subclinical hypothyroidism. The baby's thyroid function normalized within a month after starting thyroxine replacement therapy. During this period, her FT4 range remained within the normal reference range.

**Figure 1:**
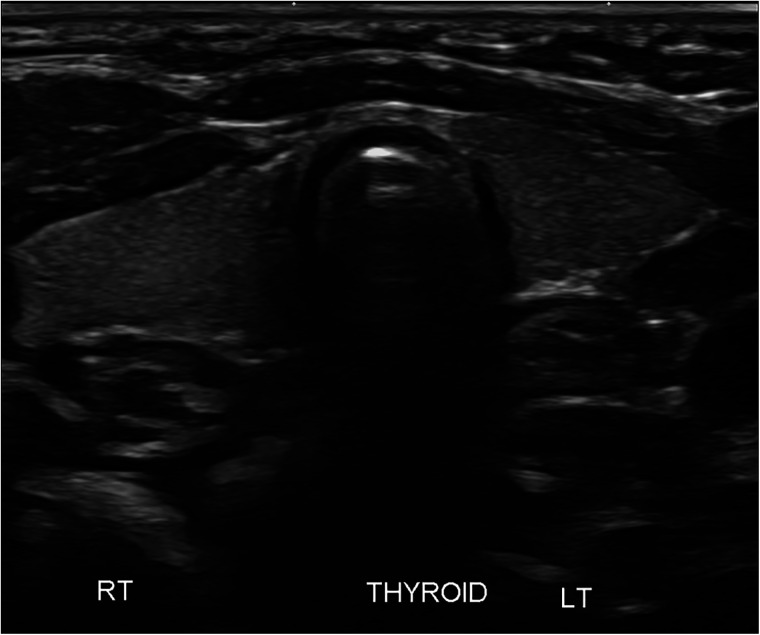
Ultrasound of the neck showing clear presence of the thyroid gland in its normal anatomical location.

**Figure 2:**
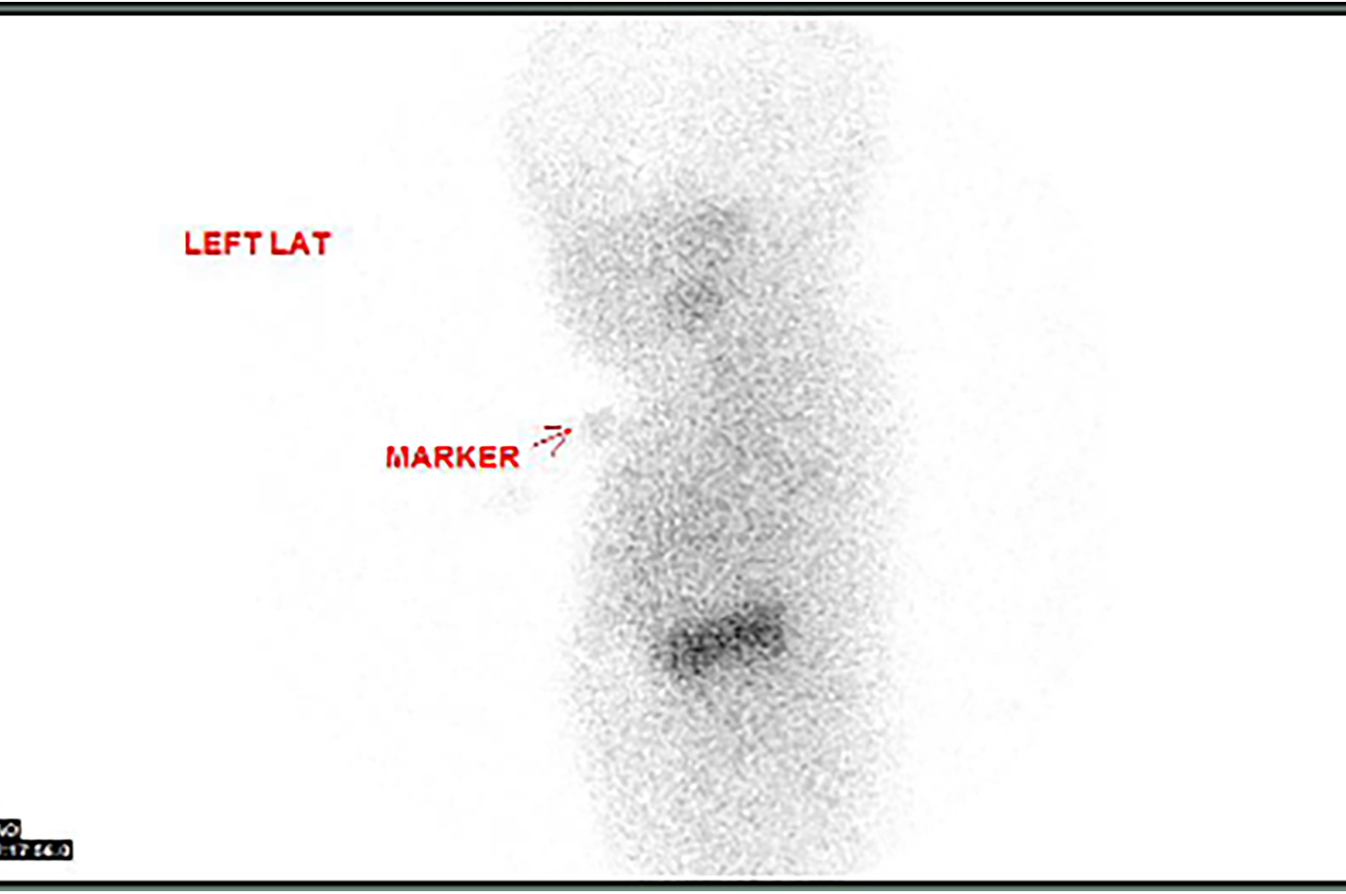
Failure of the radionuclide thyroid scan (Tc-99m pertechnetate) to detect the thyroid gland in the expected location.

An ultrasound of the thyroid gland at 6 weeks of life showed the presence of the thyroid gland in its normal anatomical location (Figure 1). A radionuclide thyroid scan (Tc-99m pertechnetate) performed on the same day failed to demonstrate the thyroid gland (Figure 2). This unique feature (i.e., the presence of thyroid glands in ultrasound but failure to locate the thyroid glands by radionuclide scans) was reported as unlikely to be due to dyshormonogenesis. A repeat radionuclide thyroid scan at 6 months of age continued to show an absence of tracer uptake by the thyroid gland.

The child received thyroxine hormone (maximum dosage: 18.75 μg and 12.5 μg alternate day) with normalization of thyroid function. The treatment was continued until 18 months of age to cover the critical phase of neurodevelopment (6–8). We opted to discontinue thyroxine as her thyroid function remained normal with a relatively low dose of thyroxine (1.9 mg/kg/day based on her weight of 8.5 kg). Thyroxine replacement was then stopped without a gradual reduction, with regular and close monitoring of thyroid function. There were no further derangements of thyroid function, and the child remained well with no side effects. Her growth and development are normal at her current age of 2 years 9 months old. Repeat thyroid function tests following discontinuation of thyroxine showed normal values appropriate for age. Table 1 presents the timeline of her thyroid function tests and doses of thyroxine and scans.

**Table 1:** Timeline of thyroid function tests, reference values, treatments, and monitoring.

Time	TSH (mIU/L)	Reference value (mIU/L)	FT4 (pmol/L)	Reference value (pmol/L)	Comments
Birth	23.96	2.20–25.00	13.5	7.5–22.0	
Day 5	18.85	0.80–5.90	19.4	17.4–57.7	Start of treatment 12.5 μg/day
Day 21	24.98	0.80–5.90	13.0	9.5–17.8	Increase to 18.75 and 12.5 μg on the alternate day^a^
Day 33	12.63	0.80–5.90	13.7	9.5–17.8	Same dose
Week 6	5.60	0.80–5.90	14.9	9.5–17.8	Same dose
Week 7	Technetium scan and thyroid ultrasound scan				
Week 15	2.14	0.70–4.17	14.5	10.5–18.8	Same dose
Week 17	Technetium scan				
Month 9	0.98	0.70–4.17	14.0	10.5–18.8	Same dose
Month 15	0.52	0.70–4.17	14.1	10–14.3	Same dose
Month 18	2.70	0.70–4.17	13.5	10–14.3	Off thyroxine
Month 19	4.81	0.70–4.17	12.1	10–14.3	Off thyroxine
Month 23	3.23	0.70–4.17	12.9	10–14.3	Off thyroxine
Month 27	3.47	0.70–4.17	14.0	10–14.3	Off thyroxine

^a^ 18.75 μg on Monday, Wednesday, Friday, and Sunday; 12.50 μg on Tuesday, Thursday, and Saturday.

### Family perspectives

The family has expressed no concerns about the child. The parents are particularly happy to share their case history to help others learn from their experience. The family had been compliant with the medication and monitoring of thyroid function after the discontinuation of thyroxine therapy.

## Discussion

We report a case of transient congenital hypothyroidism in a child of a mother who received vinblastine monotherapy for Hodgkin lymphoma during the third trimester of pregnancy.

In this neonate, the thyroid gland was visualized in its normal anatomical location via an ultrasound scan, thereby ruling out thyroid agenesis and ectopic thyroid gland as causes of congenital hypothyroidism. There was no uptake of the radioactive tracer by the thyroid gland on two occasions, therefore further excluding thyroid dyshormonogenesis as the underlying diagnosis. In addition, the absence of family history and repeatedly normal thyroid function tests in the mother ruled out maternal antibody-mediated hypothyroidism as the potential cause. Of note, thyroid dysgenesis and thyroid dyshormonogenesis account for nearly 70% and 30% of all cases of persistent congenital hypothyroidism, respectively (9).

Chemotherapeutic agents given during pregnancy can variably cross the placenta depending on their physiochemical properties, such as molecular weight, lipophilia, ionization at physiological pH, and plasma protein binding (10). Vinblastine is highly protein-bound (99%) and has a high molecular weight (811 g/mol), which contributes to its relatively low placental transfer (11). In a baboon model (*n* = 9) of transplacental transfer, 18.5 ± 15.5% of the maternal concentration of vinblastine was detected in the simultaneously taken fetal blood (11), thus supporting the notion that vinblastine could have contributed to the derangement in the thyroid function observed in this particular infant.

Cancer associated with pregnancy is an uncommon occurrence; however, its incidence has been increasing in recent years (12, 13). About 1 in 1,000 pregnancies are affected by cancer (14), with breast cancer, cervical cancer, leukemias, lymphomas, and melanoma comprising 70%–80% of pregnancy-associated cancers (10). Hodgkin lymphoma is the third (14) or fourth (15) most common cancer during pregnancy. It is generally believed that lymphoma diagnosed during pregnancy is often in an advanced stage at the time of presentation (15). In Hodgkin lymphoma, combination chemotherapy in the form of MOPP (mechloretamine, vincristine, procarbazine, prednisone) or ABVD (doxorubicin, bleomycin, vinblastine, dacarbazine) is commonly used. Vinblastine is one of the most effective drugs for Hodgkin lymphoma (4) and can be used as monotherapy to achieve disease control while limiting potential toxicity to the fetus.

Recent evidence suggests that cancer treatment during pregnancy is safe for the vast majority of neonates. In a large population-based study from Italy, Esposito et al. found no adverse neonatal outcome among 27 mothers who were exposed to chemotherapy during pregnancy (14). Capozza et al. followed up 37 newborns born to 36 mothers with pregnancy-associated cancers up to 1 year post-delivery (16). They reported no difference in neonatal outcomes between those exposed to *in utero* chemotherapy during the second and third trimesters and those who were not exposed (16).

Antineoplastic agents such as targeted therapies and immunotherapies are associated with thyroid dysfunctions in 20%–50% of patients (17). Primary hypothyroidism is the most common thyroid-related side effect, although thyrotoxicosis and other effects on thyroid-stimulating hormone secretion and thyroid hormone metabolism have also been described (17). In a study of relapsed-free survivors of childhood cancers (age 6–30 years), Çağlar et al. reported that 25% (29 out of 116) of the patients who had been exposed to vinca alkaloids developed hypothyroidism (odds ratio: 0.1, 95% CI: 0.01–1.1) (18).

The molecular mechanisms by which chemotherapeutic agents cause thyroid disorders are varied and poorly understood (19). In an *in vitro* mouse thyroid model, vinblastine has been shown to inhibit TSH-induced endocytosis and the release of thyroid hormones (20), probably due to structural disruption of microtubules in the follicle cells (21). Other proposed mechanisms include the inhibition of colloid phagocytosis by thyroid cells and the secretion of thyroid hormones by vinblastine (22). It is difficult to ascertain whether these mechanisms resulted in hypothyroidism in this newborn.

In Singapore, the reported incidence of congenital hypothyroidism screening is about one in 2,350 live births, with a female preponderance (sex ratio 2:1) (23). The cutoff value is based on cord serum TSH levels, set at ≥25 IU/L since 2000 (23). In this patient, the cord TSH value was at the upper level of normal, prompting us to repeat the thyroid function test. Within the Singapore population, among the patients with congenital hypothyroidism, a technetium isotope study of the thyroid gland revealed ectopia (abnormal location) in 52% of the cases, eutopia (normal location) in 33% of the cases, and agenesis (absent) in 10% of the cases (23). In this patient, the technetium isotope study could not show the thyroid gland (presumably absent) despite having been detected by ultrasound.

### Strengths and limitations

We describe the case following the CARE guidelines. We have a sufficient follow-up period after discontinuing thyroxine therapy. From our review of the existing literature, this has never been reported previously. However, we acknowledge some limitations. We report a particular case highlighting an association without establishing a direct link between exposure and effect. Vinblastine levels in the blood of the mother and infant were not assessed, and this measurement is not routinely performed in clinical practice and is unavailable for measurement in clinical settings. We would like to highlight other plausible reasons for a negative technetium scan. The scan was performed after the baby had been on thyroxine replacement therapy for more than 7 days, and the baby's TSH levels at birth were not very high. Both of these factors can lead to false negative results (24).

However, this report highlights the need for increased vigilance in testing for hypothyroidism in neonates born to mothers who received vinblastine or vincristine (a related agent) during pregnancy. Screening and diagnostic tests for congenital hypothyroidism are simple and widely available. Prompt treatment with replacement thyroxine therapy can prevent the long-term adverse neurodevelopmental sequelae associated with unrecognized congenital hypothyroidism.

## Data Availability

The original contributions presented in the study are included in the article/**Supplementary Material**; further inquiries can be directed to the corresponding author.
